# Transcatheter mitral valve replacement in the degenerated mitral valve prosthesis implanted in the Dacron-collar-neo-mitral-ring due to severe mitral annulus calcification

**DOI:** 10.1093/ehjcr/ytaf476

**Published:** 2025-09-27

**Authors:** Dragan Opacic, Tanja Katharina Rudolph, Lech Paluszkiewicz, Lech Hornik, Tomasz Gilis-Januszewski

**Affiliations:** Clinic for Cardiac Surgery, Heart and Diabetes Center NRW, Ruhr-University Bochum, Medical School OWL Bielefeld University, Georgstraße 11, Bad Oeynhausen 32545, Germany; Clinic for General and Interventional Cardiology, Heart and Diabetes Center NRW, Ruhr-University Bochum, Medical School OWL Bielefeld University, Georgstraße 11, Bad Oeynhausen 32545, Bad Oeynhausen 32545, Germany; Clinic for Cardiac Surgery, Heart and Diabetes Center NRW, Ruhr-University Bochum, Medical School OWL Bielefeld University, Georgstraße 11, Bad Oeynhausen 32545, Germany; Clinic for Cardiac Surgery, Heart and Diabetes Center NRW, Ruhr-University Bochum, Medical School OWL Bielefeld University, Georgstraße 11, Bad Oeynhausen 32545, Germany; Clinic for Cardiac Surgery, Heart and Diabetes Center NRW, Ruhr-University Bochum, Medical School OWL Bielefeld University, Georgstraße 11, Bad Oeynhausen 32545, Germany

**Keywords:** Mitral annulus calcification, Transcatheter mitral valve replacement, Neo-mitral ring, Mitral valve, Case report

## Abstract

**Background:**

Mitral annulus calcification (MAC) presents significant challenges in mitral valve (MV) surgery. We previously introduced a technique using a tubular Dacron prosthesis to create a neo-mitral ring, enabling prosthetic MV implantation while avoiding extensive MAC debridement. However, concerns remain regarding future valve degeneration.

**Case summary:**

A 60-year-old female with severe aortic valve (AV), tricuspid valve (TV), and MV disease, extensive MAC, and right coronary artery stenosis, along with terminal renal failure on chronic dialysis, underwent complex cardiac surgery. She received an AV replacement, MV replacement using our neo-mitral ring, TV repair, and an aorto-coronary bypass. Despite postoperative complications, she was discharged after 59 days.

Three years later, after a kidney transplant, she returned 3.5 years post-surgery with prosthetic MV degeneration. A transapical transcatheter MV replacement was performed as a ‘valve-in-valve’ procedure. The perioperative course was mostly uneventful, except for sinus arrest requiring a pacemaker. She was discharged after 16 days and remained stable for 24 months without further interventions.

**Discussion:**

Severe MAC restricts MV prosthesis implantation due to reduced orifice size. Our neo-mitral ring technique enables implantation of a sufficiently large prosthetic MV in an intra-atrial position, preserving the left ventricular outflow tract. This facilitates future transcatheter valve interventions, offering an effective long-term solution for patients with severe MAC and complex comorbidities.

Learning points

1. Creating a neo-mitral ring using a tubular Dacron prosthesis allows for the implantation of a sufficiently large mitral valve prosthesis above severe mitral annulus calcification, avoiding extensive debridement and associated risks.
2. The intra-atrial position of the valve allows for safe subsequent interventional valve procedures by removing the risk of left ventricular outflow tract obstruction. However, this position also limits the transseptal approach.
3. With ongoing advancements in treatment approaches, increasingly complex cases demand a multidisciplinary approach and innovative thinking. Close collaboration between several specialties, using both interventional and surgical means, ensures the most appropriate treatment.

## Introduction

Mitral annulus calcification (MAC) presents a formidable challenge in mitral valve (MV) surgery.^1^ Our recent publication introduces an innovative approach tailored for these high-risk patients.^2^ In brief, we have created a neo-mitral ring using a tubular Dacron prosthesis, into which a suitably sized prosthetic MV is implanted. The Dacron collar is sutured just above the calcified ring circumventing the need for extensive and risky MAC debridement. However, persistent pathophysiological factors, such as chronic kidney failure, disrupted calcium metabolism, and other chronic diseases, raise concerns regarding future calcification of the newly implanted bioprosthetic valves.^3^

## Summary figure

MAC, mitral annulus calcification; MPV, mitral prosthetic valve; TMVR, transcatheter mitral valve replacement

**Figure:**
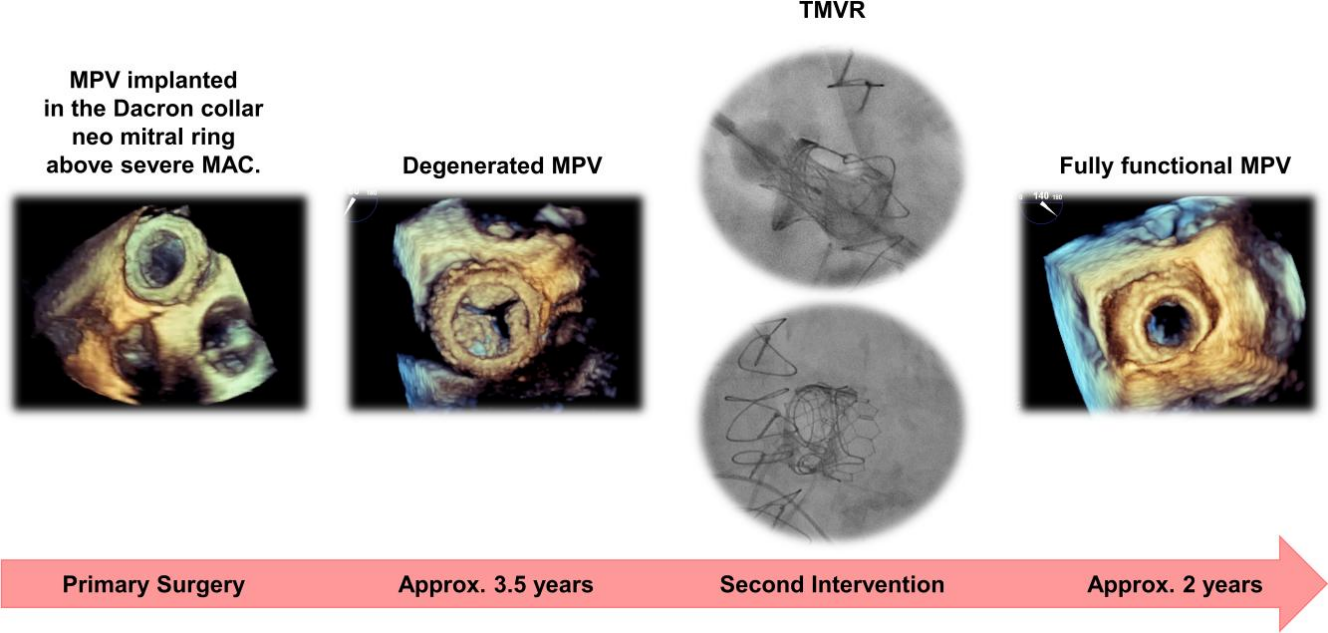


## Case presentation

A 60-year-old female patient underwent surgery for severe combined stenotic and regurgitant disease of both the aortic valve (AV) and MV, along with significant tricuspid valve (TV) regurgitation and right coronary artery stenosis. Both AV and MV showed severe calcification, with calcific bars extending towards adjacent structures. The patient also suffered from end-stage renal disease and had been on chronic dialysis for 8 years, complicated by uremic pericarditis resulting in severe pericardial adhesions. Additionally, she had paroxysmal atrial fibrillation and a history of multiple transient ischaemic attacks.

She underwent AV replacement with 23 mm Carpentier-Edwards Perimount valve, (Edwards Lifesciences, Irvine, CA) and an enlargement of the aortic root. Mitral valve replacement was performed using a 27 mm Carpentier-Edwards Perimount Magna Mitral Ease valve (Edwards Lifesciences, Irvine, CA) implanted within a neo-mitral ring constructed from a 36 mm Hemashield vascular graft prosthesis (Maquet Holding BV & Co KG, Rastatt, GER). The graft was cut slightly longer than the mitral prosthesis struts, allowing it to sit entirely above the native annulus. This positioning enabled implantation of a sufficiently large valve, reducing the risk of prosthesis–patient mismatch. The graft was secured above the annulus with felt-reinforced interrupted sutures and a continuous running suture to minimize paravalvular leak. Subsequently, the mitral valve prosthesis was implanted within the vascular graft.

**Figure 1:**
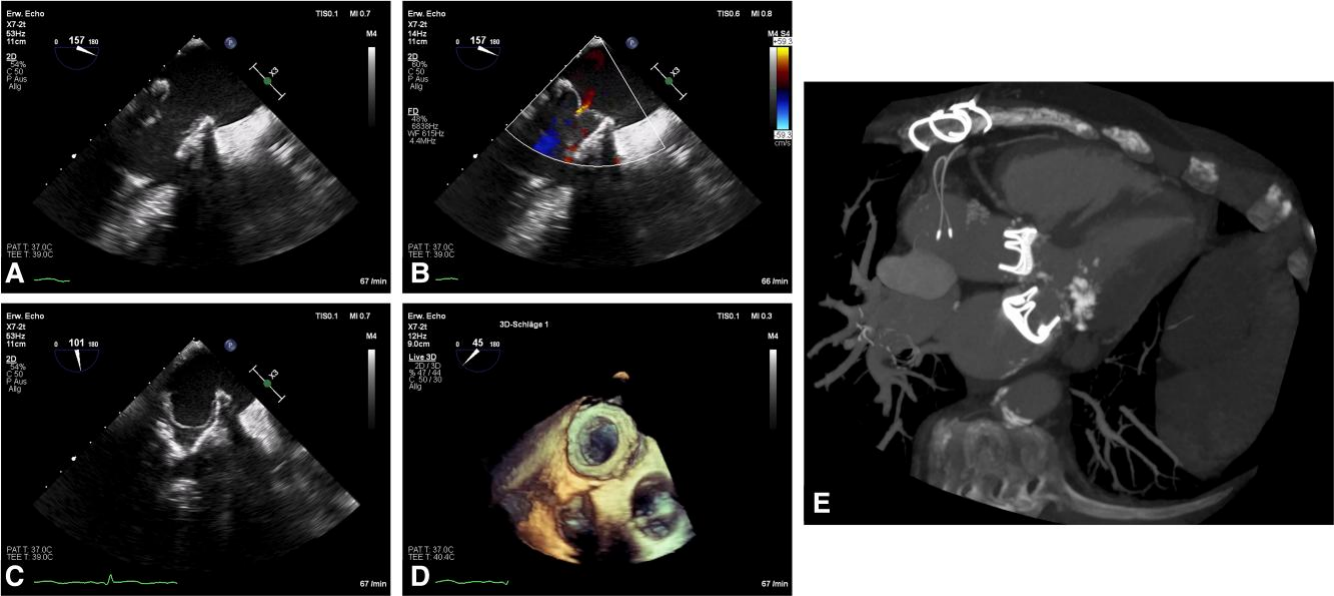
Echocardiographic findings depicting the intra-atrial implantation of a Dacron collar carrying a mitral bioprosthetic valve (*A–D*). Computed tomography scan showing the prosthetic aortic valve in its usual position, and the intra-atrially placed MPV above a severely calcified mitral annulus (*E*). MPV, mitral bioprosthetic valve; MAC, mitral annulus calcification; APV, prosthetic aortic valve.

Tricuspid valve repair was completed with a 34 mm Edwards MC3 Tricuspid ring (Edwards Lifesciences, Irvine, CA), alongside aorto-coronary venous bypass to the right coronary artery. Postoperative recovery was complex, requiring intra-aortic balloon pump support, surgical revision for tamponade, extracorporeal life support, prolonged ventilation with tracheostomy, gastrointestinal bleeding, and lymph fistula. The patient was discharged after a 59-day hospitalization. Postoperative echocardiographic findings are shown in *Figure 1A–D*.

**Figure 2:**
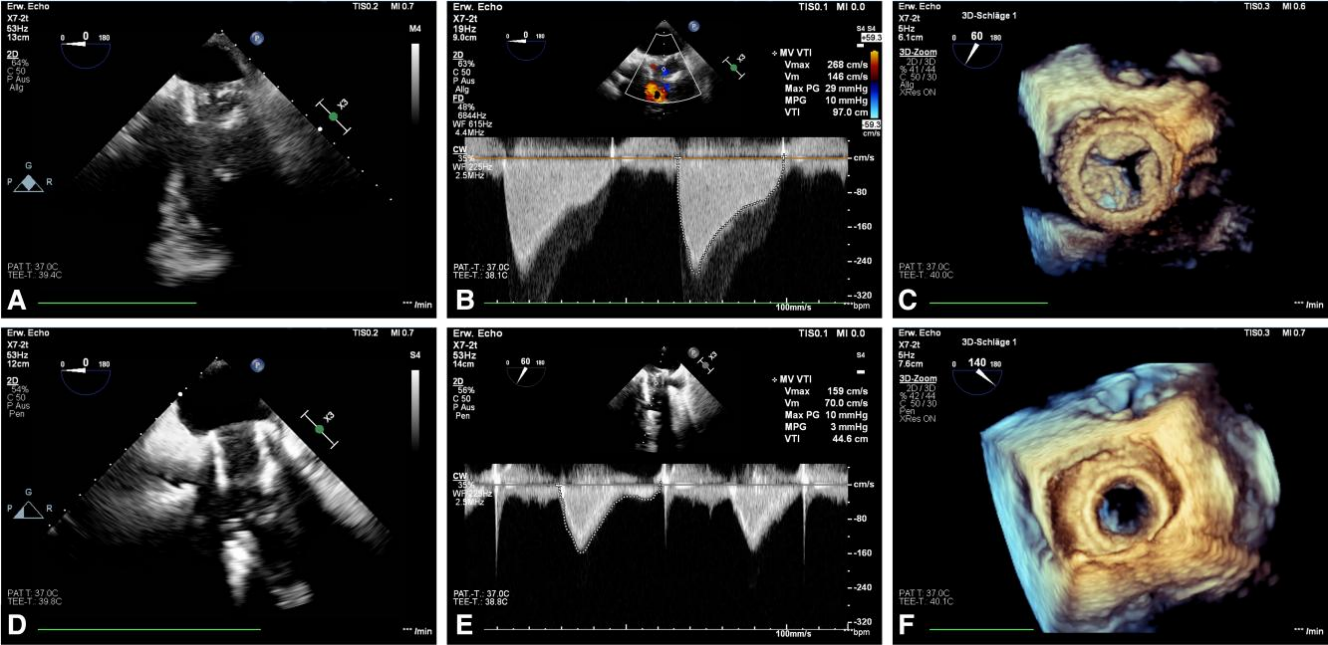
Echocardiographic findings depicting a degenerated bioprosthetic valve with a mean pressure gradient of 10 mmHg and a peak velocity of 268 cm/s without any visible paravalvular leakage. (*A–C*) Echocardiographic findings showing the full mobility of the newly implanted transcatheter mitral valve with complete leaflet motion and a major reduction of the mean pressure gradient to 3 mmHg and a peak velocity of 159 cm/s without any visible paravalvular leakage (*D–F*).

Three years later, the patient underwent successful kidney transplantation. However, progressive degeneration of the MV prosthesis was observed—likely due to chronic renal disease—leading to severe stenosis with a mean gradient of 10 mmHg and no paravalvular leak (*Figure 2A–C*). Given the elevated surgical risk, the patient was offered transcatheter MV replacement (TMVR) as ‘valve-in-valve’ procedure.

Preoperative planning was performed using 3mensio Structural Heart software (Pie Medical Imaging, Maastricht, The Netherlands) and recommendations from the Valve in Valve Mitral App (ViV Mitral).^4^ Due to the intra-atrial position of the prosthetic valve and its proximity to the atrial septum, a transseptal approach was not feasible, necessitating a transapical approach (*Figure 1E*).

**Figure 3:**
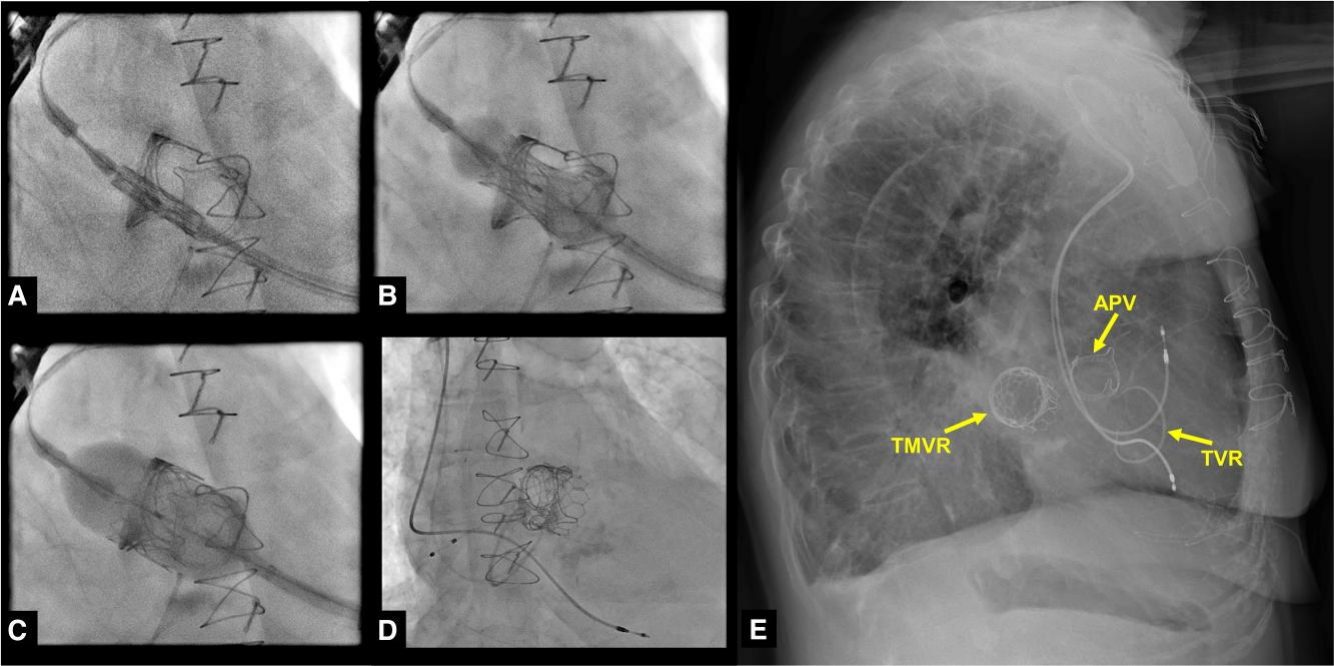
Several frames of the intraprocedural radioscopy depicting the slow and complete transapical placement of the 26 mm Sapien Ultra valve (Edwards Lifesciences, Irvine, CA) in the old mitral valve prosthesis (*A–D*). Lateral radiography showing the contours of the prosthetic aortic valve, tricuspid valve ring, and newly implanted Sapien Ultra valve as a ‘valve in valve’ in the mitral position (*E*). APV, prosthetic aortic valve; TVR, tricuspid valve ring; TMVR, transcatheter MV replacement.

The procedure involved a standard transapical access through a small left thoracotomy.^5^ Under fluoroscopy, the mitral valve was cannulated, and a 26 mm Sapien Ultra valve (Edwards Lifesciences, Irvine, CA) was implanted within the degenerated prosthesis. Key procedural steps are presented in intraoperative fluoroscopy images (*Figure 3A–D*). Post-implantation assessment revealed a mean gradient of 3 mmHg and no paravalvular leakage (*Figure 2D–F*). Preoperative and postoperative echocardiographic findings, together with fluoroscopic imaging of the implantation procedure, are shown in the accompanying video (Video 1).

The patient was extubated in the operating room and transferred awake to the intensive care unit. Recovery was largely uneventful, except for implantation of a pacemaker due to sinus arrest. She was discharged 16 days post-operatively and remained clinically stable without further cardiac intervention at 24-month follow-up. A lateral chest X-ray is shown in *Figure 3E*.

## Discussion

One of the main challenges in severe MAC is the implantation of an adequately sized valve, especially when avoiding radical debridement. Creating a neo-annulus above the MAC enables safe placement of a large prosthesis in the intra-atrial position, reducing operative risk and avoiding damage to fragile calcified structures. Moreover, this approach minimizes the risk of left ventricular outflow tract (LVOT) obstruction, which commonly complicates TMVR in patients with preserved anterior mitral leaflets.^6,7^ This is particularly relevant in patients with small or hypertrophied ventricles or coexisting AV stenosis.

Yoon *et al*.^7^ reported that patients with severe MAC undergoing TMVR had the highest 30-day and 1-year mortality rates (35% and 63%), largely due to LVOT obstruction. By contrast, valve-in-valve procedures yielded better survival rates. Similarly, slightly improved outcomes for MAC patients were observed in a retrospective analysis of data from the Society of Thoracic Surgeons/American College of Cardiology Transcatheter Valve Therapy Registry, with the most favourable results again seen in valve-in-valve procedures. The poor outcomes of TMVR in patients with severe MAC underscore the need to consider a surgical approach in this high-risk population.

Surgical treatment of MAC remains a well-known challenge in cardiac surgery.^8^ Principally, two strategies have been described: the ‘respect’ approach, in which calcifications are left in place, and the ‘resect’ approach, involving debridement of the calcified annulus.^1^ The ‘resect’ technique is technically demanding and carries a high risk of severe complications, such as atrioventricular groove disruption, which can lead to uncontrollable intraoperative bleeding. Conversely, the ‘respect’ approach avoids debridement but often limits the ability to implant an adequately sized prosthesis, increasing the risk of prosthesis–patient mismatch.

Several authors have described valve implantation in the intra-atrial position.^9–11^ However, when a rigid prosthesis is sutured directly to the thin atrial wall, it may cause intraoperative or delayed atrial wall tears, resulting in catastrophic bleeding or significant paravalvular leakage. By contrast, using a large, flexible Dacron collar as a neo-mitral ring allows the transition from the rigid prosthesis to occur within the sturdy synthetic material, rather than the fragile atrial tissue. Additionally, constructing the neo-mitral ring with the prosthesis before valve implantation allows reinforcement of the suture line between the atrial wall and the Dacron collar. This further reduces the risk of atrial wall tears and paravalvular leaks.

From a lifelong valve management perspective, our approach enables implantation of a large prosthesis within a Dacron collar, avoiding MAC debridement and reducing the risk of future LVOT obstruction during TMVR.

One important consideration is the need for anticoagulation. Because of low blood flow around the Dacron collar, strict anticoagulation is necessary to prevent thrombus formation. Additionally, the intra-atrial position of the prosthesis limits the feasibility of future transseptal interventions, such as TMVR, left atrial appendage (LAA) occlusion, or catheter ablation. Therefore, if indicated, LAA closure or atrial fibrillation ablation should be considered during the initial surgery.

## Conclusion

Severe MAC remains a condition often considered a contraindication for surgery, leading to denial of treatment for some patients. Here, we illustrate the case of an extremely ill patient who, despite significant challenges and a daunting diagnosis, received successful treatment, significantly prolonging life.

## Lead author biography


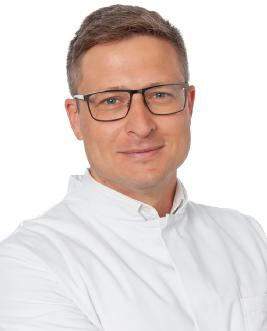


Dragan Opacic graduated from the Faculty of Medicine at the University of Belgrade. He earned his PhD as a Marie Skłodowska Curie Fellow at Maastricht University. Since 2017, he has been training in cardiac surgery at the Heart and Diabetes Center North Rhine-Westphalia, Bad Oeynhausen, and recently became a consultant surgeon.

## Supplementary Material

ytaf476_Supplementary_Data

## Data Availability

No new data were generated or analysed in support of this case report.
